# Correction to “Expression and Function of Meflin in Human Endometrial Decidualization”

**DOI:** 10.1002/rmb2.70103

**Published:** 2026-09-26

N. Inoue , Y. Tsukui , A. Morita , et al., “Expression and Function of Meflin in Human Endometrial Decidualization,” Reproductive Medicine and Biology 25, no. 1 (2026): e70048, 10.1002/rmb2.70048.PMC1305198041947913

In Section 2.3, “(3) medium with E2, progesterone (P4; 1 μg/mL; Sigma Aldrich), and N6,2′‐O‐ dibutyryl cyclic AMP (cAMP; 0.5 mM; Sigma‐ Aldrich), referred to as dibutyryl cAMP (EPA) [2, 3].” was incorrect. It should read: “(3) medium with E2, progesterone (P4; 1 μg/mL; Sigma Aldrich), and N6,2′‐O‐ dibutyryl cyclic AMP (cAMP; 0.5 mM; Sigma‐ Aldrich), referred to as EPA [2, 3].”

In Section 2.7, the description of plasmid construction was incorrect in part and contained inaccuracies regarding the primer table reference, the second‐step assembly procedure, and the control vector. It should read: “A plasmid encoding ISLR was constructed via multi‐step cloning. A pEX‐A2J2 vector containing EcoRI–ISLR cDNA (without the stop codon)–8×Gly–SalI was obtained from Eurofins Genomics (Tokyo, Japan). Both the pEX‐A2J2‐ISLR‐8×Gly and pCMV‐MycC vectors were digested with EcoRI‐HF and SalI‐HF (New England Biolabs, Ipswich, IL, USA) and ligated using T4 DNA ligase to generate pCMV‐MycC containing ISLR‐8×Gly. Assembly was confirmed by DNA sequencing (Eurofins Genomics) using the primers listed in Table S2.

The ISLR‐8×Gly‐MycC insert and the pDsRed‐Monomer‐Hyg‐N1 backbone excluding the DsRed‐Monomer coding region, except for the start codon, were amplified by high‐fidelity PCR using the primers listed in Table S2 and assembled using NEBuilder HiFi DNA Assembly (E2621X; New England Biolabs). The resulting constructs were verified by DNA sequencing (Eurofins Genomics) using the primers listed in Table S2.

The original pDsRed‐Monomer‐Hyg‐N1 vector was used as a control. iESCs were transfected with plasmids using ViaFect (Promega, Madison, WI, USA) and selected with 100 μg/mL hygromycin. Overexpression was confirmed by PCR and western blotting.”

We apologize for these errors.

